# The Effect of Preoperative Medication Intervention on Early Postoperative Cognitive Function in Elderly Patients With Poor Sleep Quality

**DOI:** 10.1155/anrp/5588639

**Published:** 2026-08-19

**Authors:** Chao Xiong, Wenhu Zhai, Rui Deng, Yanhua Peng, Xinwei Liu, Xianjie Zhang

**Affiliations:** ^1^ Department of Anesthesiology, Deyang People’s Hospital, Deyang, Sichuan, China, scu.edu.cn

## Abstract

To investigate the impact of preoperative zolpidem administration on early postoperative cognitive function in elderly patients with poor sleep quality, elderly patients undergoing gastrointestinal surgery with sleep disorders were enrolled and randomly allocated to two groups. The experimental group received 5 mg of oral zolpidem at bedtime for three consecutive nights preoperatively, whereas the control group was given a placebo. Data collected included patients’ sleep quality after medication, demographic characteristics, intraoperative parameters, and the incidence of postoperative delirium (POD). Results showed that zolpidem effectively improved preoperative sleep quality in elderly patients. However, the preoperative administration of zolpidem did not yield a statistically significant reduction in the incidence of POD in this cohort, likely attributable to inadequate statistical power. Preoperative zolpidem intervention did not significantly reduce the incidence of POD in this population. Logistic multivariate regression analysis indicated that early POD was correlated with patient age, blood transfusion, postoperative pain, and the frequency of intraoperative hypotensive episodes; however, no significant association was observed between preoperative sleep quality and the recovery of early postoperative cognitive function. In conclusion, preoperative zolpidem intervention can safely and effectively enhance sleep quality in elderly patients undergoing gastrointestinal surgery. However, the observed effect size was smaller than anticipated (Cohen’s *h* = 0.15), and the confidence interval for the difference in delirium incidence included both clinically meaningful benefit and harm. Therefore, we cannot draw definitive conclusions about whether short‐term improvement in preoperative sleep quality reduces the incidence of early POD. These findings should be considered exploratory and require validation through larger, adequately powered multicenter prospective studies with objective sleep measures and longer intervention durations.

## 1. Introduction

Insomnia is a prevalent sleep disorder affecting approximately 15% of the global population and ranks as the second most common mental health issue [1]. Chronic sleep deprivation may result in diminished alertness and reactivity, impaired cognitive functioning, low mood, and reduced attention, among other adverse effects [2]. Sleep disorders are particularly common among older adults, with their prevalence increasing with age. A study revealed that over 50% of individuals aged 60 years and above experience insomnia, while the diagnostic rate for insomnia ranges between 12% and 20% [3]. Through the investigation of sleep patterns in elderly populations, it has been established that chronic sleep disturbances are significantly associated with cognitive decline, mental health disorders, and an elevated risk of cardiovascular diseases [4]. Notably, Liu S et al. demonstrated that prolonged sleep disorders contribute to a more rapid deterioration in cognitive function, thereby highlighting a critical link between sleep quality and cognitive health [5].

Perioperative neurological dysfunction, encompassing postoperative delirium (POD) and postoperative cognitive dysfunction [6], represents a multifaceted condition. A significant proportion of elderly patients develop POD following surgery, which is associated with an elevated risk of long‐term sequelae [7]. These health challenges are of growing significance within the current sociodemographic landscape, as they are intricately linked to increased mortality rates, extended hospitalization durations, and sustained cognitive deterioration [8]. The intricate pathophysiological mechanisms underlying POD are underpinned by two primary factors: the vulnerability of elderly patients and the potential toxicity of general anesthesia and surgical interventions [9]. Sleep disorders are intricately linked to the incidence and duration of POD in elderly patients, and adequate sleep enhances the physiological resilience of elderly patients and helps maintain their stress‐balancing capacity [10]. Preoperative sleep disturbances have been shown to be associated with the development of POD [11]. However, due to insufficient emphasis on preoperative sleep management in elderly patients and their limited ability to communicate effectively, a significant number of patients with preoperative sleep disorders remain untreated, thereby increasing their susceptibility to postoperative cognitive dysfunction.

Therefore, this clinical study was designed to investigate the epidemiological characteristics of preoperative low sleep quality among elderly patients undergoing general surgery and to determine whether proactive pharmacological intervention for elderly patients with poor sleep quality prior to gastrointestinal surgery could enhance their postoperative cognitive function.

## 2. Methods

### 2.1. Study Design and Oversight

This was a randomized, double‐blind, placebo‐controlled trial conducted at the People’s Hospital of Deyang City. Participants were recruited between November 1, 2024, and April 30, 2025, with the final participant completing the follow‐up on May 3, 2025. The study received ethical approval from the Ethics Committee of the People’s Hospital of Deyang City (approval number: 2024–04–084‐K01) and was retrospectively registered in the Chinese Clinical Trial Registry on June 5, 2025 (registration number: ChiCTR2500103794). Written informed consent was obtained from all participants or their legal guardians. The study complied with all applicable institutional and national regulatory requirements. This study was conducted in accordance with the Consolidated Standards of Reporting Trials (CONSORT) guidelines for randomized controlled trials.

### 2.2. Participants

Elderly patients scheduled for elective gastrointestinal surgery at Deyang People’s Hospital from November 2024 to April 2025 will be recruited. Upon making their hospitalization appointments, the Pittsburgh Sleep Quality Index (PSQI) scoring scale will be administered to evaluate their sleep quality. A PSQI score of ≥ 7 will be defined as indicative of poor sleep quality, and the prevalence of poor sleep quality among elderly patients will be calculated.

Inclusion criteria were as follows: (1) age of 65 years or older; (2) American Society of Anesthesiologists (ASA) physical status classification of Grade III or lower; (3) PSQI > 7; (4) patients scheduled for elective gastrointestinal surgery.

Exclusion criteria were as follows: (1) long‐term use of sleep‐enhancing, psychotropic, or opioid medications; (2) ASA > III, patients with poorly controlled severe cardiovascular or cerebrovascular diseases (history of cerebrovascular accident, myocardial infarction, or unstable angina within the past 3 months, or preoperative blood pressure ≥ 180/110 mm Hg); (3) patients with allergies to any drugs involved in this study; (4) preoperative cognitive impairment; (5) patients with significant hepatic or renal insufficiency; (6) body mass index (BMI) < 18.5 or BMI > 26.9; (7) patients planned for intensive care unit (ICU) admission preoperatively or requiring unplanned ICU admission postoperatively; (8) Severe respiratory dysfunction or obstructive sleep apnea syndrome; (9) Myasthenia gravis.

Termination criteria for the study were as follows: (1) patients who request withdrawal from the study and refuse to participate in further follow‐up; (2) intolerance to the study drug or occurrence of severe adverse drug reactions; (3) other circumstances where the investigator determines that termination of the study is necessary for the patient’s safety or best interest (the administration of sedatives and sleep aids during hospitalization and similar clinical situations.).

### 2.3. Study Implementation and Data Collection

This randomized, double‐blind, parallel‐group trial adopted individual‐level randomization to allocate eligible participants to the experimental group or the control group at a 1:1 allocation ratio. Randomization was performed by an independent statistician using the PLAN procedure in SAS Version 9.4, with block randomization employing variable block sizes of 4–6. Allocation concealment was maintained via a centralized, web‐based randomization system; group assignments were disclosed to investigators only after participant enrollment was complete. An independent Data Monitoring Committee (DMC) conducted quarterly reviews of baseline characteristic balance to safeguard against allocation bias. Standardized mean differences (SMDs) for all baseline variables between the two groups were below 0.1, confirming adequate comparability across arms. To ensure allocation concealment, the generated random sequence was sealed in sequentially numbered, opaque, tamper‐evident envelopes before the initiation of participant recruitment. Envelopes were prepared and stored by an independent research assistant who was not engaged in clinical assessments. When a participant was confirmed to meet all inclusion criteria and none of the exclusion criteria, the corresponding sequentially numbered envelope was opened on‐site, and the allocation result was immediately recorded. The allocation sequence was verified at the midpoint of recruitment (after approximately 50% of participants were enrolled) by an independent statistician who compared the distribution of baseline characteristics between groups to ensure proper randomization implementation. No deviations from the planned allocation ratio or evidence of selection bias were detected. Double blinding was maintained throughout the trial. The trial interventions (experimental and control agents) were prepared in identical appearance (same shape, color, size, packaging, and labeling) by an independent pharmacy to ensure that participants, investigators, outcome assessors, and data analysts were all unaware of the group assignments. The blinding code was kept in a locked file by the statistician and was only to be broken in the event of a medical emergency or after the completion of all data collection and statistical analysis.

Elderly patients with poor sleep quality were screened for cognitive function using the Mini‐Cog assessment scale to exclude individuals with preoperative cognitive dysfunction. Eligible patients were randomly allocated into a control group (*n* = 148) and an experimental group (*n* = 148). The interval between hospital admission and surgery for patients ranged from 3 to 4 days. To ensure optimal medication management, the intervention for the experimental group consisted of oral administration of 5 mg of zolpidem tartrate tablets once daily for three consecutive days, starting 3 days prior to surgery. The control group received placebo treatment. All surgeries were conducted under total intravenous anesthesia using propofol and remifentanil. The anesthesia protocol is as follows: induction is achieved with sequential administration of sufentanil 0.3 mcg/kg, etomidate 0.3 mg/kg, and cisatracurium 0.2 mg/kg. Anesthesia is maintained with a continuous infusion of propofol at 4–8 mg/kg/h and remifentanil at 0.1–0.2 mcg/kg/min, supplemented by intermittent boluses of cisatracurium as needed. When the patient’s mean arterial pressure (MAP) decreases by more than 20% from the baseline value, 0.1 mg of phenylephrine is administered, and active interventions such as volume expansion are initiated. For postoperative analgesia, intravenous oxycodone 0.1 mg/kg is administered 30 min prior to the anticipated end of surgery. Benzodiazepines are not included in the regimen. Patient‐controlled analgesia (PCA) is provided using sufentanil as the primary agent, with nonsteroidal anti‐inflammatory drugs as adjunctive therapy. No additional analgesics or sedative‐hypnotic agents are administered during the postoperative period in the ward.

On the morning of the operation, researchers blinded to the group assignments evaluated the patients’ sleep quality over the preceding three days using the Numerical Rating Scale (NRS), where 0 indicated the poorest sleep quality and 10 the best. They also assessed for any severe adverse drug reactions. Additionally, they documented the patients’ demographic characteristics (e.g., gender, age, and BMI), comorbidities (e.g., coronary heart disease, diabetes, hypertension, and chronic obstructive pulmonary disease), history of alcohol consumption, hypoalbuminemia (serum albumin less than 35 g/L), anesthesia duration during the operation, intraoperative hypotension (the MAP decreased by 20% or more relative to the preoperative baseline value) occurrences, blood loss, fluid infusion volume, blood transfusion and other surgical details. POD in elderly patients predominantly occurs within the first 3 days following surgery. The follow‐up period of this study was therefore established by referencing the time frame protocols employed in prior relevant studies [12, 13]. On the first and third postoperative days (10:00 a.m. and 3:00 p.m.), dedicated follow‐up personnel, who were also blinded to the group assignments, used the Chinese version of the 3‐min Confusion Assessment Method (3D‐CAM) to assess the patients for delirium, and utilized the NRS to evaluate the patient’s pain status. All follow‐up personnel received standardized training to ensure consistency in assessment procedures. The Kappa coefficient for interassessor consistency was 0.72 (95% CI: 0.58–0.86), indicating a high level of consistency between the two assessors.

### 2.4. Outcomes

The primary outcome measure was the occurrence of early POD in patients. Secondary outcome measures included the incidence of poor sleep quality in elderly patients before surgery, the efficacy of zolpidem tartrate tablets in improving sleep quality in elderly patients, and correlation analyses between preoperative factors (e.g., sleep quality) and early POD.

### 2.5. Sample Size Estimation

Based on prior research, the incidence of POD in elderly patients undergoing gastrointestinal surgery varies between 8.2% and 54.4% [14]. Given that patients with sleep disorders exhibit a higher prevalence of POD, we established the delirium incidence rate (p2) for the control group at 30%, while hypothesizing that the delirium incidence rate (p1) for the experimental group would be 15%. With a type I error (*α*) set at 0.05 and a type II error (*β*) at 0.2, alongside a sample size ratio (*k*) of 1:1 between the experimental and control groups, the calculated sample size for each group is 118. Accounting for an anticipated dropout rate of 20%, the adjusted sample size requirement increases to at least 148 participants for both the experimental and control groups.

However, the actual observed effect size was smaller than anticipated (Cohen’s *h* = 0.15 vs. the expected *h* = 0.33). The observed difference in delirium incidence between groups was 6.08% (95% CI: −2.9%–15.1%), which includes both clinically meaningful benefit and harm. This wide confidence interval (CI) reflects the uncertainty in our estimate and suggests that the initial sample size calculation was based on an overestimated effect size from previous literature. Future studies should consider using more conservative effect size estimates and potentially larger sample sizes or multicenter designs to obtain more precise effect estimates.

### 2.6. Statistical Analysis

**FIGURE 1:**
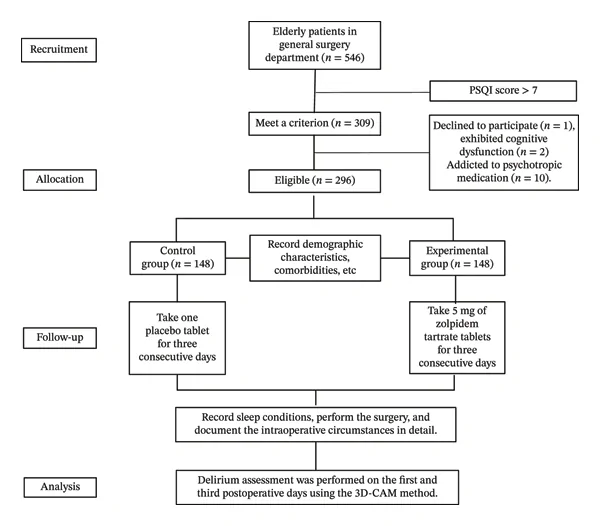
Consolidated standards of reporting trials (CONSORT) flow diagram for the study protocol.

Data analysis was performed using SPSS 25.0 software. Normality was assessed using the Shapiro–Wilk test. Continuous variables that followed a normal distribution were presented as mean ± standard deviation (SD), whereas those that deviated from normality were summarized as median and interquartile range. For comparisons among three groups, one‐way ANOVA or the Kruskal–Wallis test was employed, depending on the data distribution. For pairwise comparisons between two groups, the *t*‐test or Wilcoxon rank‐sum test was utilized, as appropriate. Binary variables were described using frequency and percentage and compared using the chi‐square test or Fisher’s exact test, based on the assumptions of the tests. To investigate the relationship between influencing factors and delayed postoperative neurocognitive recovery, binary logistic multivariate regression analysis was conducted. The results of the logistic regression analysis were reported as odds ratios (ORs) with their corresponding 95% CIs. *p* value less than 0.05 was considered indicative of statistical significance (Figure 1).

Although a formal, detailed statistical analysis plan (SAP) was not prospectively registered, the primary and secondary outcomes, along with the main analytical approaches, were specified in the study protocol submitted to the Ethics Committee. All analyses presented in this manuscript were conducted according to these prespecified objectives. However, some post hoc analyses (including stratified analyses and estimation of observed effect sizes with CIs) were conducted after data collection and are clearly labeled as exploratory.

Regarding multiple comparisons, no formal adjustment was applied to the secondary outcomes and subgroup analyses, as these were considered exploratory in nature. The primary outcome (incidence of POD) was tested at the two‐sided *α* = 0.05 level without adjustment, as it was the sole primary endpoint. For secondary outcomes and exploratory analyses, we report unadjusted *p* values with their corresponding CIs, and readers should interpret these findings with appropriate caution given the potential for type I error inflation.

Missing data were minimal in this study. Less than 2% of participants had missing values for any baseline or outcome variables. Participants with missing primary outcome data (*n* = 0) were excluded from the primary analysis. For baseline characteristics, missing values were present in < 1% of cases for BMI (*n* = 2) and education level (*n* = 1), and these participants were excluded from analyses involving these specific variables (complete‐case analysis). No imputation was performed given the low proportion of missing data. A sensitivity analysis comparing complete cases with all randomized participants showed no substantial differences in baseline characteristics or outcomes.

All cases with missing data were excluded during the data collection phase, ensuring that the analytical dataset comprised only complete observations. Consequently, no imputation or other missing‐data handling procedures were required.

All primary analyses were conducted following the intention‐to‐treat (ITT) principle, which included all randomized participants in the groups to which they were originally assigned, regardless of protocol adherence or withdrawal. This approach preserves the benefits of randomization and provides a conservative estimate of treatment effects. A per‐protocol analysis was also performed as a sensitivity analysis, including only participants who completed the study protocol as specified.

Post hoc analyses, including stratified analysis by comorbidity severity and estimation of observed effect sizes with CIs, were conducted after data collection to explore potential explanations for the primary outcome results. These analyses are clearly labeled as exploratory and should be interpreted with caution.

## 3. Results

### 3.1. Epidemiological Characteristics of Poor Preoperative Sleep Quality in Elderly Patients: A Comprehensive Analysis

A total of 546 individuals were included in the analysis. Among them, 44 patients had been using sleep‐aid medications over an extended period, resulting in a medication prevalence of 8.1%. Currently, 309 individuals exhibit poor sleep quality, with an incidence rate of 56.6%. The Pearson correlation coefficient between sleep disorders and age is approximately 0.167, indicating a moderate positive correlation between sleep disorders and age, although the association remains relatively weak. Among elderly patients, there is a notable trend of high incidence of poor sleep quality prior to surgery, yet the rate of intervention remains low.

### 3.2. Zolpidem Tartrate Can Safely and Effectively Improve Sleep Quality in Elderly Patients

To evaluate the efficacy and safety of zolpidem tartrate in elderly patients with sleep disorders, all enrolled patients were randomly assigned to receive either placebo or zolpidem tartrate. The results demonstrated a significant improvement in the preoperative NRS sleep assessment scale in the experimental group compared to the control group (6.70 ± 1.15 vs. 5.23 ± 1.39, *p* < 0.001). Apart from mild adverse events such as visual disturbances and next‐day drowsiness reported in a small number of patients, no serious adverse reactions were observed. Zolpidem tartrate may therefore be considered safe and effective for short‐term use in elderly patients with sleep disorders.

### 3.3. General Demographic Information and Surgical Characteristics of the Study Population

A total of 309 patients with poor sleep quality were enrolled in the study. Among them, one patient declined to participate, two patients exhibited preoperative cognitive dysfunction, and 10 patients had a history of long‐term psychotropic drug use. Ultimately, 296 patients completed the study. The SMD was employed to evaluate the balance of baseline characteristics between the two groups. No statistically significant differences were observed between the control group and the experimental group in terms of gender, age, BMI, education level, ASA classification, duration of surgery and anesthesia, fluid infusion volume, blood loss volume, blood transfusion volume, hypoalbuminemia, postoperative pain, alcohol consumption history, or baseline cognitive function (*p* > 0.05). However, among patients with comorbidities, the proportion was significantly lower in the control group compared to the experimental group (70.3% vs. 81.8%, *p* = 0.021, Table 1).

**TABLE 1:** Comparison of demographic data and general conditions of patients.

Category	Control group (*n* = 148)	Experimental group (*n* = 148)	*p*
Preoperative PSQI score, mean ± SD	5.23 ± 1.39	6.7 ± 1.15	< 0.001
Age (years), mean ± SD	75.01 ± 5.98	75.16 ± 5.81	0.828
Gender, frequency (%)			0.058
Male	67 (45.27%)	51 (34.46%)	
Female	81 (54.73%)	97 (65.54%)	
BMI (kg/m2), mean ± SD	22.47 ± 3.53	22.18 ± 3.10	0.386
Education (years), frequency (%)			0.64
≧ 3	72 (48.65%)	66 (44.59%)	
< 3	76 (51.35%)	82 (55.41%)	
ASA			0.978
II	13 (8.78%)	12 (8.11%)	
III	126 (85.14%)	127 (85.81%)	
IV	9 (6.08%)	9 (6.08%)	
Comorbidities, frequency (%)	104 (70.3%)	121 (81.8%)	0.021
The severity of comorbidities, mean ± SD	1.12 ± 0.91	1.16 ± 0.84	0.691
History of alcohol consumption, frequency (%)	36 (24.3%)	28 (18.9%)	
Preoperative Mini‐cog, mean ± SD	2.91 ± 0.62	2.93 ± 0.61	0.78
Duration of surgery (mins), mean ± SD	170.51 ± 31.06	176.93 ± 34.87	0.096
Duration of anesthesia (mins), mean ± SD	185.61 ± 30.97	192.09 ± 34.94	0.092
Infusion quantity (mL), mean ± SD	1289.19 ± 463.66	1249.49 ± 437.74	0.449
Bleeding (mL), mean ± SD	87.74 ± 123.65	67.97 ± 83.59	0.108
Blood transfusion ratio, frequency (%)	12 (8.11%)	14 (9.46%)	0.681
Pain score (postoperative Day 1), mean ± SD	3.95 ± 0.88	3.77 ± 0.79	0.062
Pain score (postoperative Day 3), mean ± SD	1.67 ± 0.66	1.57 ± 0.67	0.224
Hypoalbuminemia (< 35 g/L), frequency (%)	45 (30.4%)	44 (29.7%)	0.899

### 3.4. Short‐Term Preoperative Sleep Interventions Appear to Have No Significant Effect on Reducing the Incidence of Postoperative Cognitive Dysfunction

The overall incidence of POD was 22.30% in the experimental group and 16.22% in the control group. Although the incidence in the experimental group was slightly higher than that in the control group, the difference was not statistically significant (*p* = 0.185). On the initial day following surgery, the overall incidence of delirium was 14.53%, with no statistically significant difference observed between the two groups (13.51% vs. 15.54%, *p* = 0.621). On postoperative Day 3, the overall incidence of delirium decreased to 10.14%, and there remained no statistically significant difference in the incidence of delirium between the two groups (10.81% vs. 9.46%, *p* = 0.700) (Table 2). Univariate regression analysis was performed concurrently to examine the association between the occurrence of POD and sleep quality scores (OR = 1.08, *p* = 0.428). The results indicated that improved short‐term sleep quality was only weakly associated with the incidence of early POD.

**TABLE 2:** Comparison of postoperative delirium occurrence in patients.

POD	Control group (*n* = 148)	Experimental group (*n* = 148)	SUM (*n* = 296)	Statistic	*p*
Day 1				0.245	0.621
No	128 (86.49%)	125 (84.46%)	253 (85.47%)		
Yes	20 (13.51%)	23 (15.54%)	43 (14.53%)		
Day 3				0.148	0.700
No	132 (89.19%)	134 (90.54%)	266 (89.86%)		
Yes	16 (10.81%)	14 (9.46%)	30 (10.14%)		
Cumulative incidence				1.760	0.185
No	124 (83.78%)	115 (77.70%)	239 (80.74%)		
Yes	24 (16.22%)	33 (22.30%)	57 (19.26%)		

### 3.5. Age, Blood Transfusion, Postoperative Pain, and Intraoperative Hypotension Are Significant Risk Factors for the Development of POD in Elderly Patients Undergoing Gastrointestinal Surgery

To further investigate the risk factors for POD in elderly patients undergoing gastrointestinal surgery, we performed a univariate regression analysis to identify potential risk factors and subsequently integrated these findings with established risk factors reported in the literature to conduct a forward multivariate regression analysis. This analysis considered POD and its associated factors in conjunction with currently recognized risk factors. The regression analysis indicated an events‐per‐variable (EPV) ratio of less than 10, suggesting inadequate statistical power and a heightened risk of parameter estimation bias. To assess overall model calibration, the Hosmer–Lemeshow test was performed, yielding a nonsignificant *p* value of 0.626, consistent with adequate goodness‐of‐fit. In light of the limited sample size and potential for separation or small‐sample bias, Firth’s penalized‐likelihood logistic regression was applied to obtain more robust and nearly unbiased coefficient estimates. The results indicated no significant correlation between preoperative sleep quality and the early onset of POD. However, patient age (OR = 1.17 [1.10–1.25], *p* < 0.001), intraoperative blood transfusion (OR = 14.88 [5.21–46.35], *p* < 0.001) and moderate to severe postoperative pain (OR = 10.24 [3.44–41.52], *p* < 0.001) and intraoperative hypotension (OR = 1.10 [1.02–1.18], *p* = 0.011) were significantly associated with the occurrence of POD. No substantial evidence was found to support a significant relationship between preoperative sleep quality and POD (Table 3, Table 4).

**TABLE 3:** Multivariate logistic analysis of postoperative delirium.

Dependent variable	Independent variable	B	Standard error	Z	*p*	OR [95%CI]
POD	constant	−18.967	3.197	−5.933	< 0.001	
	Age	0.167	0.034	4.865	< 0.001	1.18 [1.1,1.26]
	ASA ≥ III	0.280	0.675	0.415	0.678	1.32 [0.35,4.97]
	Preoperative sleep score	0.148	0.138	1.077	0.282	1.16 [0.89,1.52]
	Blood transfusion	2.871	0.579	4.958	< 0.001	17.66 [5.68,54.94]
	Intraoperative hypotension	0.097	0.037	2.587	0.01	1.1 [1.02,1.19]
	Moderate to severe postoperative pain	2.575	0.675	3.817	< 0.001	13.13 [3.5,49.28]
	Hypoproteinemia	0.512	0.398	1.288	0.198	1.67 [0.77,3.64]
	History of alcohol consumption	0.724	0.436	1.660	0.097	2.06 [0.88,4.84]
	Low level of education	−0.053	0.414	−0.128	0.898	0.95 [0.42,2.14]

**TABLE 4:** Multivariate logistic regression analysis of delirium with Firth’s penalized likelihood correction.

Dependent variable	Independent variable	B	Standard error	Z	*p*	OR[95% CI]
POD	Age	0.156	0.032	4.885	< 0.001	1.17 [1.10–1.25]
	ASA ≥ III	0.209	0.626	0.334	0.744	1.23 [0.37–4.77]
	Preoperative sleep score	0.136	0.130	1.053	0.303	1.15 [0.89–1.50]
	Blood transfusion	2.700	0.541	4.989	< 0.001	14.88 [5.21–46.35]
	Intraoperative hypotension	0.090	0.035	2.561	0.011	1.10 [1.02–1.18]
	Moderate to severe postoperative pain	2.326	0.596	3.901	< 0.001	10.24 [3.44–41.52]
	Hypoproteinemia	0.493	0.376	1.310	0.203	1.64 [0.76–3.48]
	History of alcohol consumption	0.693	0.411	1.686	0.102	2.00 [0.87–4.57]
	Low level of education	−0.058	0.391	−0.149	0.884	0.94 [0.43–2.10]

The extremely high odds ratio for blood transfusion (OR = 14.88) warrants cautious interpretation. This elevated estimate likely reflects the relatively small number of patients who received blood transfusions, resulting in wide CIs and potential instability of the point estimate. Such extreme OR values in logistic regression can occur when there is separation or near‐separation in the data, where the outcome is rare in one category of the predictor. While blood transfusion is clearly associated with delirium in our cohort, the precise magnitude of this association should be confirmed in larger studies with more transfusion events.

Given that patients in the experimental group exhibited a higher burden of comorbidities compared to those in the control group, a stratified analysis was performed to minimize potential confounding effects of comorbidities on study outcomes. Comorbidities were categorized into four levels based on both the number of coexisting conditions and associated clinical manifestations, with higher categories indicating greater comorbidity severity. Within‐stratum effects and the overall pooled stratified effects of delirium between the two groups were assessed according to these comorbidity categories. The homogeneity test for the odds ratio across strata showed consistency (*p* = 0.955), and the Mantel–Haenszel method yielded a combined stratified odds ratio of 1.68 (*p* = 0.103). These results indicate that comorbidities did not act as a confounding factor influencing the difference in delirium incidence between the groups (Table 5).

**TABLE 5:** Stratified analysis of comorbidities and the incidence of delirium.

Stratification	POD	Control group	Experimental group	OR[95% CI]	*p*
0	Positive	4 (8.89%)	4 (13.79%)	1.06 [0.89,1.26]	0.780
	Negative	41 (91.11%)	25 (86.21%)		

1	Positive	5 (10.42%)	11 (13.92%)	1.04 [0.91,1.19]	0.763
	Negative	43 (89.58%)	68 (86.08%)		

2	Positive	13 (27.66%)	12 (44.44%)	1.32 [0.89,1.91]	0.225
	Negative	34 (72.34%)	15 (55.56%)		

3	Positive	3 (37.50%)	6 (46.15%)	1.16 [0.56,2.42]	0.999
	Negative	5 (62.50%)	7 (53.85%)		

Mantel–Haenszel				1.68 [0.90,3.14]	0.103

## 4. Discussion

This clinical study investigated the epidemiological characteristics of preoperative sleep deficiency in elderly patients within the research area. The findings indicated a high prevalence of poor sleep quality among elderly patients prior to surgery, with insufficient cognitive and intervention strategies. The hypnotic drug zolpidem tartrate was demonstrated to be safely and effectively applicable to elderly patients for short‐term improvement of preoperative sleep quality; however, such improvement did not appear to enhance postoperative cognitive function nor reduce the incidence of POD in elderly patients.

Sleep disorders are widely recognized as a significant public health concern, with profound implications for cognitive function and overall well‐being. High‐quality sleep plays an indispensable role in maintaining physical health [15]. Studies have shown that the prevalence of sleep disorders among elderly individuals exceeds 50%, with insomnia rates ranging from 15% to 20% [3], findings that align with our research. Our investigation revealed that 56.6% of elderly patients experienced poor sleep quality prior to surgery. However, it is important to note that we solely utilized the PSQI to evaluate sleep quality. Given the complexity of diagnosing sleep disorders, this assessment does not comprehensively represent the incidence of such conditions, including insomnia, frequent awakenings, or difficulty maintaining sleep. Furthermore, sleep quality is influenced by living environment and demographic distribution characteristics, which may account for variations across studies [16]. Additionally, our findings indicate that in this region, the high prevalence of poor sleep quality among elderly presurgical patients is exacerbated by inadequate awareness and intervention, significantly impacting the health and well‐being of a substantial elderly population.

According to the statistics on the annual consumption of sleeping pills in China provided by the National Medical Products Administration, it is evident that the Chinese population consumes approximately 339 million doses of sleeping pills annually, with zolpidem being the most widely prescribed hypnotic agent [17]. Meanwhile, research findings regarding the association between benzodiazepine treatment and cognitive decline in elderly patients remain inconclusive. Questions persist concerning the causal relationship and the influence of confounding factors on the interpretation of these studies [18]. The effects of zolpidem tartrate tablets, a nonbenzodiazepine hypnotic drug, on postoperative cognitive function in elderly patients have yet to be fully elucidated [19]. Zolpidem has been shown to subjectively improve sleep quality without impairing next‐day performance. Furthermore, repeated administration does not appear to induce tolerance to its effects on sleep latency or sleep quality, nor does it exacerbate adverse effects on task performance [20]. Therefore, we selected zolpidem tartrate as the preoperative sleep intervention drug for investigation. In this study, we observed that a 3‐day zolpidem intervention prior to surgery significantly enhanced the sleep quality of elderly patients, with no severe adverse effects and excellent tolerability. A low dose of zolpidem tartrate tablets can be safely and effectively administered to elderly patients.

Research has established a bidirectional relationship between sleep quality and cognitive function in older adults [21]. Additionally, a multi‐cohort study investigating the association between cognitive function and sleep quality revealed that cognitive decline in middle‐aged and older individuals correlates with worsening sleep quality [22]. The influence of subjective sleep quality on cognitive function in older adults is sequentially mediated by anxiety and depressive symptoms. Consequently, various interventions targeting the improvement of sleep quality in older adults may enhance both mood and cognitive performance [23]. Drug intervention remains the predominant method for managing sleep disorders in elderly patients. Huo S et al. [24] demonstrated in their study on the effects of drug intervention on sleep quality in Alzheimer’s disease (AD) patients that eszopiclone can significantly enhance both sleep quality and cognitive function in elderly individuals with AD and sleep disturbances. Additionally, hypnotic medications appear to improve cognitive impairment and alleviate depressive mood in patients who have undergone brain tumor resection [25]. Nevertheless, the impact of hypnotic drugs on cognitive function remains a subject of debate. A meta‐analysis revealed that the use of zolpidem and benzodiazepines is linked to an elevated risk of dementia and AD [26]. In a study examining the effects of zolpidem on nocturnal sleep and overnight episodic memory enhancement, it was demonstrated that zolpidem significantly improves episodic memory function in patients over the course of a night and is beneficial for memory cognition [27]. To investigate whether preoperative pharmacological intervention could enhance postoperative cognitive function in elderly patients, zolpidem tartrate was administered orally for three consecutive days prior to surgery to improve sleep quality. Additionally, to reduce the potential confounding effects of anesthetic drugs and techniques, total intravenous anesthesia primarily based on propofol was utilized. Propofol‐based total intravenous anesthesia has minimal influence on postoperative cognitive function and sleep quality in elderly patients [28]. Through systematic research, it was determined that the overall incidence of POD in elderly patients undergoing gastrointestinal surgery was 19.26%, with an incidence rate of 14.53% on the first day and 10.14% on the third day postsurgery. These findings are consistent with the conclusions reported in prior studies [29]. However, short‐term enhancement of sleep quality in elderly patients before surgery using zolpidem did not appear to significantly reduce the incidence of POD in this population. The lack of difference in the incidence of POD between the two groups may be attributable to multiple factors: First, the duration of drug administration was relatively brief, and as a result, underlying symptoms such as anxiety and depression associated with sleep disorders and physical conditions were not substantially alleviated. As research has confirmed, preoperative quetiapine administration improves patients’ sleep quality and cognitive function [30], and adjunctive interventions such as antidepressant therapy may further enhance postoperative cognitive outcomes. Short‐term improvements in sleep quality seem insufficient to enhance postoperative cognitive function in patients. Second, the effects of zolpidem on cognitive function remain a subject of ongoing debate in the scientific community [20, 31, 32]; zolpidem itself may exert adverse effects on the cognitive function of elderly patients, as supported by previous research indicating that long‐term use may compromise cognitive preservation in this demographic. Consequently, it remains challenging to clearly delineate the interplay between the drug’s inherent effects and its role in improving sleep quality on the cognitive function of elderly patients. Third, postoperative factors such as pain, intraoperative hypotension, and blood transfusion may have overwhelmed any potential beneficial effects of the preoperative sleep intervention. Our multivariate analysis identified these factors as strong independent predictors of POD, with particularly large effect sizes for blood transfusion and postoperative pain. These potent perioperative stressors may have masked any modest protective effect of preoperative sleep improvement.

There are various risk factors for POD in elderly patients, including age, educational attainment, type of surgery, preoperative anxiety and depression status, postoperative pain, hypoproteinemia, and the presence of severe comorbidities [33]. Numerous studies have demonstrated that preoperative sleep disorders serve as independent risk factors for postoperative cognitive impairment. In our multivariate logistic regression analysis, which incorporated multiple variables such as preoperative sleep quality, we identified that patient age, blood transfusion, postoperative pain, and the frequency of intraoperative hypotensive episodes were significantly associated with the incidence of POD, findings that align with prior research [14]. However, our results indicated no significant association between sleep quality and the occurrence of POD, which contrasts with previous studies. This discrepancy may be attributable to the limited time available for improving preoperative sleep quality and the complex interactions of medications on cognitive function. Further investigation appears warranted.

Our study acknowledges certain limitations and areas for improvement. First, the duration of drug intervention was relatively brief, which may have restricted our ability to observe whether short‐term improvements in sleep quality significantly influence postoperative cognitive function in elderly patients. Second, the follow‐up period was insufficiently long, and the assessment of cognitive function was somewhat limited in scope. Although the 3D‐CAM has demonstrated strong validation and operational efficiency, its sensitivity may be suboptimal for detecting subtle or fluctuating delirium presentations—particularly hypoactive delirium, which is highly prevalent among older adults. Some cases of delirium may have been missed, especially those occurring outside the assessment windows or those with atypical presentations. Additionally, we did not evaluate the duration or severity of delirium in the two patient groups. Third, the PSQI and NRS are subjective, self‐reported measures that may be susceptible to recall bias and social desirability bias. The absence of objective sleep measures such as polysomnography (PSG) or actigraphy represents a significant limitation of our study. Subjective sleep quality ratings may not accurately reflect objective sleep parameters such as sleep efficiency, total sleep time, or sleep architecture. Future studies should incorporate both subjective and objective sleep measures to provide a more comprehensive assessment of sleep quality and its relationship with postoperative outcomes. Fourth, the observed effect size (Cohen’s *h* = 0.15) was smaller than anticipated, and the 95% CI for the difference in delirium incidence between groups was wide (−2.9% to 15.1%), including both clinically meaningful benefit and harm. The wide CI indicates substantial uncertainty about the true effect of the intervention, and we cannot exclude the possibility of either a small beneficial effect or a small harmful effect. Consequently, the nonsignificant difference in delirium incidence should be interpreted with caution, as the study was not designed to detect such small effect sizes. Given these limitations, this study should be considered exploratory, and the findings regarding the relationship between preoperative sleep intervention and POD should be interpreted as hypothesis‐generating rather than confirmatory. Future multicenter studies with larger sample sizes and standardized delirium assessment protocols are warranted to confirm the potential efficacy of the intervention. Fifth, we did not perform equivalence testing, which could have provided additional insight into whether the intervention effect was statistically equivalent to no effect within a prespecified margin. Future studies should consider incorporating equivalence or noninferiority designs.

## 5. Conclusion

The prevalence of poor sleep quality among elderly patients undergoing elective surgery is relatively high. Preoperative administration of zolpidem tartrate tablets can safely and effectively enhance sleep quality in elderly patients. However, given the observed small effect size and wide CIs, we cannot draw definitive conclusions about whether short‐term improvement in sleep quality reduces the incidence of early POD in elderly patients undergoing gastrointestinal surgery. The findings regarding POD should be considered exploratory and hypothesis‐generating. This study highlights the methodological challenges in conducting preoperative intervention trials and underscores the importance of adequate statistical power, objective outcome measures, and consideration of multiple perioperative factors that may influence cognitive outcomes.

Despite the limitations, our findings highlight the importance of the relationship between sleep disorders and postoperative cognitive function, which can guide the development of targeted preventive strategies in clinical practice. Future multicenter studies with improved study design and sufficient statistical power are needed to further elucidate the risk factors and protective factors for POD in elderly patients.

Given the exploratory nature of these findings and the study’s limitations, causal claims about the relationship between preoperative sleep intervention and postoperative cognitive outcomes should be avoided. Our results should be interpreted as preliminary evidence that requires confirmation in larger, more rigorously designed studies.

## Author Contributions

Chao Xiong and Xianjie Zhang conceived and designed the study. Wenhu Zhai and Rui Deng were responsible for collecting case data. Yanhua Peng was responsible for conducting postoperative follow‐up. Chao Xiong drafted the manuscript. Xianjie Zhang completed the final revisions of the manuscript. All authors revised the manuscript and gave final approval of the version to be submitted.

## Funding

This study was supported by the Sichuan Provincial Research Center for Healthy Aging Development, Project Supporting Fund No: 24SCLN079.

## Consent

The authors have nothing to report.

## Conflicts of Interest

The authors declare no conflicts of interest.

## Data Availability

The majority of data are included in the manuscript. The integrated data are available upon request by contacting the corresponding author via email.
